# A Study of Otolaryngology Residency Match Outcomes from the National Residency Matching Program

**DOI:** 10.1002/oto2.70260

**Published:** 2026-06-16

**Authors:** Akshay Warrier, Rohan Singh, Nikita Jaiswal, Aakash Patel, Evelyne Kalyoussef, Kenneth Yan

**Affiliations:** ^1^ Department of Otolaryngology–Head and Neck Surgery Rutgers New Jersey Medical School Newark New Jersey USA; ^2^ Department of Otolaryngology–Head and Neck Surgery University of New Mexico School of Medicine Albuquerque New Mexico USA

**Keywords:** NRMP, otolaryngology, research, residency, the match

## Abstract

**Objective:**

To identify trends and evaluate key predictors of matching success in the otolaryngology residency match from 2016 to 2024.

**Study Design:**

A retrospective cohort study using data from the National Resident Matching Program (NRMP).

**Setting:**

National residency match data analysis up to March 2024.

**Methods:**

NRMP data from 2016 to 2024 were analyzed to assess trends in otolaryngology programs, positions, and applicants. Competitiveness was evaluated using the applicant‐to‐position ratio. Predictors of match success—including USMLE Step 2 scores, research productivity, Alpha Omega Alpha (AOA) membership, MD/PhD status, and medical school ranking—were compared between matched and unmatched applicants.

**Results:**

From 2016 to 2024, the number of otolaryngology programs rose from 109 to 138, with available positions increasing from 304 to 382. Applicants also increased (370‐513), raising the applicant‐to‐position ratio by 6.9%. Matched applicants had significantly higher Step 2 scores (255.2 vs 247.8, *P* < .001), more research experiences (13.94 vs 9.62, *P* < .001), and ranked more programs (13.52 vs 6.68, *P* < .001). Research output grew at an annual rate of 11.9% (*P* < .001). AOA membership, MD/PhD status, and attending a top 40 medical school were not significant predictors of matching.

**Conclusion:**

Competitiveness in otolaryngology residency has intensified, driven by rising applicant numbers and research output. While Step 2 scores remain a reliable predictor, AOA status and MD/PhD degrees do not appear to influence match success. The increasing emphasis on research may incentivize quantity over quality, highlighting the need for programs to reconsider applicant evaluation metrics.

**Level of Evidence:**

3.

Since 1952, the National Residency Match Program (NRMP) has structured and recorded residency “Match” outcomes, filling ACGME‐accredited programs using a Nobel Prize‐associated algorithm matching student and program preferences.^1^ Beyond managing the match, the NRMP also collects and reports data through annual publications like *Results and Data* and *Charting Outcomes of the Match*, providing valuable insights into trends and characteristics of matched and unmatched applicants, helping to elucidate shifts in medical education and their impact on the residency landscape.

Understanding the match process and characteristics of successfully matched applicants is crucial amid increasingly competitive specialties. Prior research in plastic surgery, neurosurgery, and orthopedic surgery has investigated NRMP data to understand this unsustainable trend. These analyses revealed a proclivity towards increased research and USMLE emphasis, offering important insights and alternatives to the current system. Suggestions have included the allocation of funding towards increased residency spots, the evaluation of emotional intelligence (correlated positively with job satisfaction and negatively with burnout), and an emphasis on personal applicant characteristics, including letters of recommendation and responses to nationally standardized interview questions.^2^, ^3^, ^4^, ^5^ While studies in the past have individually investigated increasing residency applications and applicant characteristics, there has yet to be a comprehensive analysis of match trends within otolaryngology.

As of 2024, there are 138 ACGME‐accredited otolaryngology residency programs in the United States, a number which has been steadily increasing over time. Despite this growth, the specialty continues to be extraordinarily competitive.^6^ Otolaryngology is unique in the residency match landscape as it has seen the greatest rise in applicants compared to other established surgical subspecialties, necessitating a deep investigation into the contributing factors.^7^

The timing of this study is particularly relevant in light of recent changes to the application process and applicant characteristics, such as the COVID‐19 pandemic and curriculum changes.^8^, ^9^, ^10^ In particular, the USMLE Step 1 exam was converted to pass/fail, a decision that has created a volte‐face within the residency application landscape.^11^, ^12^, ^13^ This shift emphasizes step 2 scores and variable clinical grading scales and have intensified a “publication arms race”^14^, ^15^ These changes significantly impact otolaryngology applicants. Increased competitiveness has led applicants to “shotgun” applications, applying to more programs to improve matching odds, consequently imposing financial burdens.^16^, ^17^, ^18^, ^19^ Meanwhile, residency programs have responded to the surge in applications by implementing stricter screening criteria, further compounding challenges within the application process.^18^, ^19^ A key adaptation was making otolaryngology a “high signal” specialty in 2023 so that interviews are mainly offered to applicants who signal respective programs. This categorically serves as a soft application cap while benefitting less competitive applicants and reducing the load on applicants and programs alike.^20^, ^21^ It is important to acknowledge that otolaryngology applicant numbers have not risen uniformly over the past 2 decades, but rather follow cyclical fluctuations. A substantial surge occurred between 2012 and 2015, followed by a relative decline before the current study window.^1^

This study has 2 main objectives. First, it seeks to analyze trends in the application process and residency program characteristics over time, identifying patterns that reflect the increasing competitiveness of otolaryngology residency applications. Second, it aims to explore the specific characteristics of successfully matched applicants, shedding light on the objective attributes that residency programs prioritize in their selections. This investigation is crucial amid evolving residency preferences and the growing interest in otolaryngology.

## Materials and Methods

### Data Collection

Data were collected from the *Results and Data: Main Residency Match* and *Charting Outcomes in the Match* reports for the years 2016 to 2024. From the *Results and Data* reports, information was gathered on the number of otolaryngology programs, positions offered, positions filled, and the number of applicants. Data on applicant categories included the number of US medical students who applied and matched, as well as other categories such as International Medical Graduates (IMGs), osteopathic graduates, and reapplicants.

The Charting Outcomes reports provided applicant‐specific data, including USMLE Step 1 and Step 2 scores, numbers of abstracts, presentations, and publications, Alpha Omega Alpha (AOA) membership, and the percentage from top 40 NIH‐funded schools. Step 1 means were calculated only for years with numeric scores (before 2022) and omitted from growth models after the transition to pass/fail. The NRMP defines “research experiences” as abstracts, poster presentations, and peer‐reviewed publications. Data were analyzed for US allopathic students ranking otolaryngology as their first‐choice specialty, defined as the specialty listed first or the only specialty listed. The cohort was limited to US allopathic seniors, a more homogeneous group, to avoid confounding from the heterogeneous “Independent” category. Formal IRB exemption was obtained through the Rutgers University Office of Research IRB research board.

### Data Analysis

Competitiveness in otolaryngology was assessed by calculating the applicant‐to‐position ratio and match rate, modeling after similar studies by Dossani et al and Rynecki et al.^4^, ^5^ The applicant‐to‐position ratio was defined as the number of US seniors who ranked otolaryngology as their first choice divided by the number of available residency positions. The match rate was calculated as the percentage of US seniors who successfully matched into otolaryngology relative to the total number of US seniors who ranked otolaryngology as their preferred specialty.

Trends in applicant characteristics such as USMLE scores, number of publications, and other relevant metrics were analyzed. Comparisons between matched and unmatched applicants were performed using two‐tailed *t*‐tests for continuous variables and chi‐square tests for categorical variables. Annual trends were reported as annual growth rates, evaluated with exponential regression models applying a null‐growth hypothesis, where the baseline assumption was “no change” over time. The significance of growth models was tested with an *α* of 0.05. Statistical analysis was conducted using SPSS Version 26.0 (IBM Corp.).

## Results

### Trends in Otolaryngology Programs, Positions, and Applicants

From 2016 to 2024, the number of ACGME‐accredited otolaryngology residency programs steadily increased from 109 to 138, reflecting an annual growth rate of 3.4% (95% CI: 2.7%, 4.3%; *P* < .001). The number of residency positions similarly increased from 304 in 2016 to 382 in 2024, representing an annual growth rate of 3.1% (95% CI: 2.6%, 3.6%; *P* < .001) (Table 1). This expansion in residency positions was accompanied by a rise in the total number of applicants, from 370 in 2016 to 513 in 2024. The number of US seniors who listed otolaryngology as their preferred specialty also exhibited a significant increase, despite fluctuating between 297 and 455 during this period, with an overall growth rate of 2.3% (95% CI: 1.5%, 3.2%; *P* < .001). The number of matched US seniors also showed a significant increase over the study period, rising from 272 in 2016 to 339 in 2024 (2.3%; 95% CI: 1.5%, 3.2%; *P* < .001).

**Table 1:** Trends in Residency Applications for Otolaryngology and All US Medical Students from 2016 to 2024

Otolaryngology								All specialties		
Year	No. of programs	No. of positions	Total no. of applicants	No. of preferred US seniors	No. of preferred USMD matched	Preferred application position ratio	Preferred match rate	No. of positions	No. of active applicants	Applicant to position ratio
2016	109	304	370	306	272	1.007	0.889	27,825	35,476	1.275
2017	110	305	331	298	279	0.977	0.936	28,796	35,969	1.249
2018	112	315	333	297	284	0.943	0.956	30,150	37,103	1.231
2019	120	328	462	394	308	1.201	0.782	32,108	38,376	1.195
2020	129	350	505	414	310	1.183	0.749	34,167	40,084	1.173
2021	129	350	559	448	310	1.28	0.692	35,194	42,508	1.208
2022	134	361	574	455	316	1.260	0.695	36,277	42,549	1.1729
2023	138	373	493	369	310	0.989	0.840	37,425	42,952	1.1477
2024	138	382	513	414	339	1.084	0.819	38,494	44,853	1.165
Annual growth rate % (95%)	+3.4% (2.7%, 4.3%)	+3.1% (2.6%, 3.6%)	+6.5% (2.1%, 11.1%)	+4.8% (1.0%, 8.9%)	+2.3% (1.5%, 3.2%)	+2.4% (1.5%, 3.3%)	−2.4% (−5.5%, 0.9%)	4.3% (3.8%, 4.9%)	3.1% (2.6%, 3.7%)	−1.2% (−1.6%, −0.7%)
*P*‐value	***P*** < **.001**	***P*** < **.001**	***P*** = **.009**	***P*** = **.021**	***P*** < **.001**	***P*** < **.001**	*P* = .133	***P*** < **.001**	***P*** < **.001**	***P*** < **.001**

Bold values indicate statistically significant results *P* < .05.

### Competitiveness in the Otolaryngology Match

**Figure 1:**
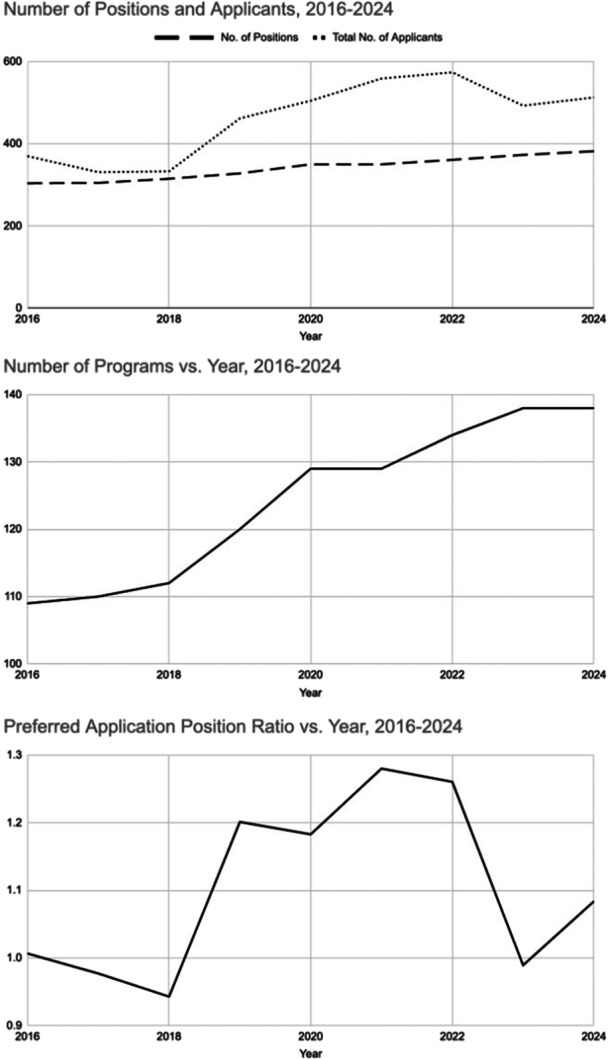
Trends in otolaryngology programs and competitiveness from 2016 to 2024.

The competitiveness of otolaryngology residency programs, as measured by the applicant‐to‐position ratio, increased significantly from 1.01 in 2016 to 1.08 in 2024 (annual growth rate of 2.4%, 95% CI: 1.5%, 3.3%; *P* < .001). While the total number of applicants grew during the study period, the match rate for US seniors exhibited no overall consistent trend, fluctuating between a high of 93.6% in 2017 to a low of 69.2% in 2021 (2.4%; 95% CI: −5.5%, 0.9%; *P* = .133) (Table 1). Otolaryngology experienced a decreased rate of growth in residency positions (3.1% annually for otolaryngology vs 4.3% across all specialties), while the applicant‐to‐position ratio for otolaryngology increased at a much faster pace (+2.4% annually) than the overall match, which actually saw a decrease of 1.2% (95% CI: −1.6%, −0.7%; *P* < .001) annually (Table 1 and Figure 1).

### Trends in Otolaryngology Residency Applicant Characteristics

Over the years, the only major characteristic of matched otolaryngology residency applicants that has significantly changed is the number of research items, which has increased at an annual growth rate of 11.9% from 2016 to 2024 (95% CI: 9.7%‐14%). Research items significantly increased for all matched applicants over the years (9.6%; 95% CI: 8.3%‐11%; *P* < .001) but not for unmatched otolaryngology applicants (0.3%; 95% CI: −2 to 9%; *P* = .182). This is consistent when analyzing all ENT applicants as a cohort, as research productivity remains the only variable that significantly changes (11.25%; 95% CI: 4.55%‐17.96%; *P* < .001) (Table 2). For applicants across all specialties, but not for otolaryngology, only Step 2 scores (annual increase of 2%; 95% CI: 0%‐4%; *P* = .002) and applications from top 40 programs (annual decrease of 0.7%; 95% CI: −1%, −0.4%) have changed significantly over the years.

**Table 2:** Comparison of Characteristics Between US Medical Graduates Matching into Otolaryngology Versus Other Specialties

	Cohort	2016 N(M) = 257, N(UM) = 33	2018 N(M) = 276, N (UM) = 12	2020 N(M) = 299, N (UM) = 96	2022 N(M) = 266, N (UM) = 112	2024 N(M) = 268, N (UM) = 54	Annual growth rate% (95% CI)	*P*‐value
Contiguous Ranked	ENT matched	12.7	14.3	13.1	13.9	13.6	0.5% (−2.0%, 3.1%)	*P* = .546
	ENT unmatched	7.5	5.7	7.1	5.9	7.2	−2.9% (−12.7%, 120.3%)	*P* = .459
	ENT combined	12.1	13.9	11.6	11.5	12.5	0.43% (−0.96%, 1.81%)	*P* = .546
	All matched^a^	11.8	12.3	12.5	13.8	13.2	1.71% (0.63%‐2.80%)	*P* = .053
USMLE Step 1 mean^b^	ENT matched	248	248	248	250	‐	−0.2% (−0.7%, 0.4%)	*P* = .404
	ENT unmatched	240	238	243	243	‐	0.2% (−0.2%, 0.6%)	*P* = .229
	ENT combined	247	247.6	246.8	247.9	‐	−0.22% (0.29%, −0.73%)	*P* = .404
	All matched^a^	233	233	234	236	‐	1.7% (0.0%, 3.5%)	*P* = .053
Step 2	ENT matched	253	254	256	257	256	0.2% (0.0%, 0.4%)	*P* = .057
	ENT unmatched	247	242	249	250	251	0.2% (−0.2%, 0.6%)	*P* = .229
	ENT combined	252.3	253.5	254.3	254.9	255.2	0.14% (−0.00%, 0.28%)	*P* = .057
	All matched^a^	245	246	247	248	250	0.2% (0.2%, 0.3%)	***P*** = **.002**
Research	ENT matched	8.4	10.4	13.7	17.2	20	11.9% (9.7%, 14.0%)	***P*** < **.001**
	ENT unmatched	6.7	5.3	9.5	11	15.6	0.3% (−0.3%, 0.9%)	*P* = .182
	ENT combined	8.2	19.2	12.7	15.4	19.3	11.25% (4.55%, 17.96%)	***P*** < **.001**
	All matched^a^	4.7	5.7	6.9	7.9	10	9.6% (8.3%, 11.0%)	***P*** < **.001**
AOA^c^	ENT matched	44.7	40.2	38.1	41	33.6	−2.8% (−6.0%, 0.7%)	*P* = .084
	ENT unmatched	15.2	8.3	21.9	18.8	16.7	5.1% (−13.5%, 27.8%)	*P* = .472
	ENT combined	41.3	38.9	34.2	34.4	30.8	−3.63% (0.49%, −7.74%)	*P* = .084
	All matched^a^	17.3	17	16.7	16.8	17.1	−0.2% (−0.9%, 0.6%)	*P* = .511
Top 40^c^	ENT matched	40.1	30.1	40.8	39.1	36.6	0.4% (−6.6%, 7.9%)	*P* = .872
	ENT unmatched	36.4	8.3	21.9	25	27.8	2.8% (−25.5%, 42.2%)	*P* = .800
	ENT combined	39.7	29.2	35.7	35.2	35.1	−1.51% (16.9%, −19.9%)	*P* = .872
	All matched^a^	32.1	31.9	31	30.8	30.5	−0.7% (−1.0%, −0.4%)	***P*** = **.007**
MD/PhD^c^	ENT matched	3.3	4.6	2.8	3.6	2.8	−2.9% (−12.7%, 8.2%)	*P* = .459
	ENT unmatched	3.3	0	2.1	0	4.1	2.7% (−63.2%, 187.2%)	*P* = .794
	ENT combined	3.3	4.4	2.7	3.2	2.3	−4.26% (7.0%, −15.5%)	*P* = .459
	All matched^a^	4.1	4	3.7	3.8	3.8	−1.0% (−2.6%, 0.6%)	*P* = .133

^a^ All matched refers to the aggregate total of all residency applications submitted to NRMP, not only restricted to otolaryngology.

^b^ Data available up until Pass/Fail Designation in 2022.

^c^ Percentage of cohort.

### Predictors of Matching into Otolaryngology

**Figure 2:**
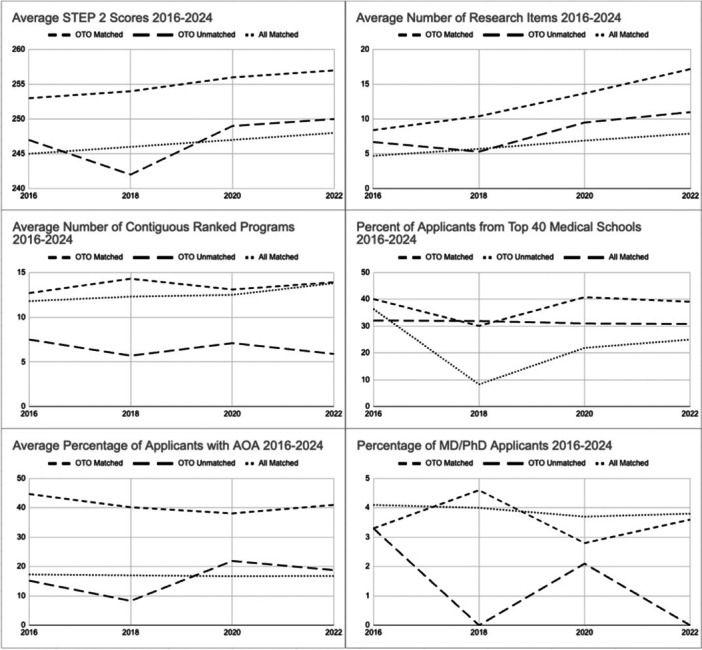
Trends in otolaryngology match predictors from 2016 to 2024.

Averaged across the years, matched applicants ranked a significantly higher number of otolaryngology programs, with a mean of 13.52 programs ranked, compared to 6.68 for unmatched applicants (*P* < .001). Additionally, matched applicants demonstrated higher USMLE Step 1 and Step 2 scores. The mean Step 1 score prior to the pass/fail designation for matched applicants was 247.4, while unmatched applicants had a mean score of 241.2 (*P* < .001). Similarly, matched applicants had a significantly higher mean Step 2 score (255.2) compared to unmatched applicants (247.8) (*P* < .001). Research activity also played a significant role, with matched applicants reporting a mean of 13.94 research experiences, compared to 9.62 for unmatched applicants (*P* < .001) (Table 3). Although other characteristics such as Alpha Omega Alpha (AOA) membership and graduation from a top 40 NIH‐funded medical school showed trends toward higher rates among matched applicants, these differences were not statistically significant. Specifically, 39.52% of matched applicants were AOA members compared to 23.88% of unmatched applicants (*P* = .126), and 37.34% of matched applicants graduated from a top 40 NIH‐funded medical school, compared to 23.88% of unmatched applicants (*P* = .186). MD/PhD status was also not a significant predictor of matching success, as both matched and unmatched groups had similar proportions of applicants with MD/PhD degrees (*P* = .39) (Table 3 and Figure 2).

**Table 3:** Predictors of a Successful Otolaryngology Match for Graduating US Medical Students who Ranked Otolaryngology as Their Preferred Specialty

	Matched	Unmatched	*P*‐value
Contiguous ranked (mean)	13.5	6.7	**<.001**
USMLE Step 1 (mean)^a^	247.4	241.2	**<.001**
USMLE Step 2 (mean)	255.2	247.8	**<.001**
Publications (mean)	13.9	9.6	**<.001**
AOA (mean %)	39.5	23.9	.126
Top 40 (mean %)	37.3	23.9	.186
MD/PhD (mean %)	3.4	1.9	.390

^a^ Data available until pass/fail designation in 2022.

## Discussion

Otolaryngology is an increasingly competitive subspecialty for residency applicants. This paper attempts to elucidate the fundamental factors driving this trend by analyzing application processes and characteristics of successfully matched residents from 2016 to 2024. Over the eight‐year period, accredited otolaryngology programs increased by 26.6% and residency positions by 26.0%, but total applicants rose by 38.6%, raising the applicant‐to‐position ratio from 1.01 to 1.08. This disparity shows that applicant interest has outpaced available positions, reflecting increased competition. For matched applicants, the number of research items was the only predictor that significantly changed over the years. Unexpectedly, AOA membership, graduation from a top 40 NIH‐funded medical school, and MD/PhD status were not significant predictors of matching success. As otolaryngology residency programs become more competitive, prospective applicants must understand which objective markers are most important to maximize their likelihood of matching.

### Understanding the Trends: The Competition

Otolaryngology's increased competitiveness can be partially attributed to the disproportionate rise in applicants compared with the number of available residency positions. Our results demonstrated that the applicant‐to‐position ratio has significantly increased over the past 8 years by approximately 2.4% annually, indicating a trend that may limit opportunities for qualified candidates (Table 1). At first glance, this pattern does not seem unique to otolaryngology; other competitive specialties, such as orthopedic surgery, have similarly experienced an increase in the number of applicants and residency positions.^22^, ^23^ However, they have not seen a significant rise in the applicant‐to‐position ratio, suggesting that these fields have aligned their growth in applicants with the expansion of training opportunities.^4^ Notably, prior literature has shown that the increase in applicant‐to‐position ratio in otolaryngology has historically been greater than in other competitive specialties such as dermatology, orthopedic surgery, and general surgery, reflecting an ongoing mismatch between the interest in otolaryngology and the available training capacity.^24^ This disparity highlights the need for more positions to prevent qualified applicants from being excluded. While more otolaryngologists are needed nationally, maldistribution across subspecialties and persistent shortages in rural areas suggest that workforce planning must address both the quantity and geographic distribution of graduates.^25^, ^26^ Addressing this issue may require a multi‐faceted approach, including increased funding for residency positions, curriculum changes, and enhanced mentorship to help applicants navigate this increasingly competitive landscape.^27^

### Understanding the Trends: Predictors of Matching

The evolving dynamics of otolaryngology residency selection reveal that objective metrics such as research output, USMLE Step 2 scores, and the number of programs ranked are emerging as pivotal predictors of matching success. Research production, in particular, has seized the attention and focus of many medical students, with its role in shaping residency outcomes becoming increasingly important, especially in a competitive field like ENT. Our study corroborates this by demonstrating a growing trend of research by residents and a greater match success rate with larger volumes of publications. This has contributed to the growing publication arms race, where medical students feel increasing pressure to publish more research articles to differentiate themselves from their peers, demonstrated across the literature.^28^, ^29^, ^30^, ^31^ This pressure can fuel burnout, encourage misrepresentation of research (“ghost‐authorship”), and deter students by reinforcing a narrow, metrics‐focused perception of otolaryngology.^31^, ^32^ This is not unique to otolaryngology; for instance, neurosurgery residency applicants have significantly increased publications annually, with similar research misrepresentation.^2^, ^33^ Prospective orthopedic surgery residents also participate in the publication arms race, with residency programs continuously raising their minimum requirement threshold.^34^ This trend is not sustainable. The average matched applicant 10 years ago had fewer publications than the average unmatched applicant now, and more medical students are redirecting time spent studying for board exams to research.^14^ Research in medical school does not strongly correlate with clinical performance. Programs should consider limiting the number of research papers on ERAS and adopting metrics that assess research quality, institutional support, and authorship trends.

The transition to a STEP 1 pass/fail system has removed one of the major grading benchmarks for residency matching and, as a result, increased emphasis on other grading metrics. Our study emphasized the importance of STEP 1 and STEP 2 scores between matched and unmatched applicants. In otolaryngology, the change to pass/fail is anticipated to increase the significance of STEP 2 scores, core clerkship grades, elective rotations, AOA and other rewards, and letters of recommendation. This places underrepresented students and international students at a disadvantage, increasing anxiety for interviews, and increasing the success rate of students from top‐ranked institutions.^11^ Similar changes are anticipated in other competitive residency programs. In neurosurgery, program directors expect the change will make objective comparisons of applicants difficult and put greater emphasis on STEP 2 scores and medical school reputation.^35^ Ob/Gyn reports similar challenges with the loss of a strong performance predictor, likely shifting emphasis to STEP 2 scores and letters of recommendation.^36^

The decline of AOA designations and variability in grading systems have shifted greater emphasis toward Step 2 scores and research output. However, Step 2 scores showed no significant change over time, likely reflecting a ceiling effect among consistently high‐performing applicants. Although matched applicants scored on average 7.4 points higher than unmatched peers, the overlapping distributions suggest that many unsuccessful applicants still achieved competitive scores. This overlap highlights the growing importance of supplemental factors such as research productivity. Furthermore, the temporary shift to virtual interviews during COVID‐19 may have amplified application numbers by lowering financial and travel barriers.

Surprisingly, factors like AOA status, MD/PhD degree, or attending top NIH‐funded schools were not significant predictors of matching success in otolaryngology. This may be due to reliance on other metrics like USMLE Step 2 scores and research output, which more directly reflect an applicant's individual academic and clinical proficiency.^30^ Although these differences did not reach statistical significance, the absolute gaps between groups (15 percentage points) suggest larger datasets may be needed to fully evaluate their predictive value. Holistic review processes now favored by many residency programs may also focus on a candidate's overall fit, commitment to the specialty, and individual achievements so as to decrease socioeconomic disparities and increase diversity and cohesiveness within the program.^37^, ^38^

### Future Research

Future research should assess the long‐term effects of the Step 1 pass/fail transition on otolaryngology and other competitive specialties, as current data remain limited. Studies are also needed to evaluate the impact of virtual interviews on match outcomes. In addition, further investigation into factors not captured here—such as letters of recommendation, personal statements, and away rotations—may clarify their role in applicant success. Including osteopathic and international graduates would provide a more comprehensive view of the match landscape. To address the escalating research arms race, the ARCS calculator proposed by Bowers et al offers a promising tool to prioritize quality over quantity.^39^ Finally, future selection strategies could consider limiting reported research items, adopting structured interview tools, or expanding holistic review frameworks that better capture applicant values, communication skills, and resilience.

### Limitations

This study has a few limitations. Data were drawn from NRMP publications, which do not capture key factors such as interpersonal skills, letters, and away rotations. Limited data points also precluded analysis of the Step 1 pass/fail transition. As a retrospective review, causation cannot be inferred. We did not report *R* ^2^ values given lack of NRMP data, and research productivity findings may be skewed; future analyses using medians and interquartile ranges may better reflect distribution.

## Conclusion

This study sheds light on the increasing competitiveness of otolaryngology residency programs, revealing the critical factors that predict matching success. Over the 8‐year study period, both the number of programs and applicants grew significantly, with the applicant‐to‐position ratio reflecting a more competitive environment. Higher USMLE Step 2 scores, research productivity, and the number of programs ranked emerged as significant predictors of matching success, while traditional markers such as AOA membership and MD/PhD status did not. These findings emphasize the overt “publication arms race” and the shifting emphasis towards Step 2 scores due to the transition of Step 1 to pass/fail grading, albeit demonstrating a possible score plateau. As the otolaryngology match becomes more competitive, applicants must adapt their strategies by focusing on high‐yield predictors of success, particularly research and academic performance. Residency programs, on the other hand, may need to re‐evaluate their selection criteria to balance the increasing applicant pool while ensuring holistic assessments of applicants and quality physicians. Thus, there is a dire need for continued research into adaptations and new formulations of the application process as the landscape of medical education evolves.

## Author Contributions

**Akshay Warrier**, project ideation, data collection, data analysis, writing; **Rohan Singh**, project ideation, data collection, data analysis; **Nikita Jaiswal**, writing; **Aakash Patel**, writing; **Evelyne Kalyoussef**, project ideation, editing; **Kenneth Yan**, project ideation, editing.

## Disclosures

### Competing interests

None.

### Funding source

None.
